# Emergency Contraception: An Updated Review

**Published:** 2011-10-17

**Authors:** M. Guida, M.L. Marra, V. Palatucci, R. Pascale, F. Visconti, F. Zullo

**Affiliations:** 1. Department of Obstetrics & Gynecology, University of Salerno, Italy

**Keywords:** emergency contraception, levonorgestrel, mifepristone, ulipristal acetate

## Abstract

Emergency contraception is a common practice now. Many categories of drugs are marketed with modifications in dosage, in combination and even in the timing of administration. Recent re-analysis suggests that there is still no uniformity of opinion on the actual mechanism of action and this has often fueled the ethical controversy. This review analyzes the most common emergency contraception drugs: levonorgestrel, mifepristone and ulipristal acetate about their action underlining that the hormonal products, when used in emergency contraception, play different roles depending on the phase of the menstrual cycle during which they are administered.This review aims to examine rigorously the most accredited literature to verify if a evidence-based uniformity of opinions has been achieved about the biological effects of hormones administered after the sexual intercourse.

## 1 Introduction

Since the second half of the last century, hormonal products have began to be used for contraceptive purposes as a social consequence of a real “cultural revolution” [1].

The role of the hormonal contraception has been crucial to foster women’s empowerment and gender equality [2,3].

Moreover, unlike other categories of drugs, the contraceptive products (generally referred to as “the pill”) have undergone continuous modifications in dosage, combination and even in the timing of drug administration – today it is administered also after sexual intercourses [4,5]. Nowadays we can state that hormonal contraception can be prescribed to healthy women of all ages [6].

Nevertheless, these progresses have initially met harsh resistances from the doctors themselves mainly for ethical and religious reasons especially when cases may occur that hormonal products would damage embryo and not only avoid conception.

This is still a highly topical issue especially when considering the so-called “emergency contraception” that makes use of hormonal products only after a sexual intercourse.

Scientific literature and international cultural debate have not yet reached a common view on the actual mechanism of action of some hormone molecules when administered after sexual intercourse. It is clear that the ethical (and thus religious) judgement cannot neglect the strict and rigorous awareness of the effects that the hormonal emergency contraception causes before and after conception, that is damage to the embryo or before the embryo starts to develop.

Bitter controversy have aroused about this delicate issue. This review wants to underline that very often there is no clear distinction between the legal aspects and the ethical aspects of this issue.

On the contrary, we deem that the legal aspect of the use of emergency contraception should be appraised according to the law in force in each country.

Ethical facets do not take into consideration the legal frameworks, indeed they need a moral evaluation. In short, it only answers the question about the possible damage that these hormones may cause on the embryo which a lot of religions consider untouchable.

It goes without saying that those who are in favour of intentional abortion (usually included in the legislation of many countries) will see no harm in emergency contraception taking place at the very early phases of the embryo development and will consider this opportunity as an improvement in the health services offered to the women who do not intend to carry on an unexpected pregnancy.

It is nonetheless true that those who do administer hormones to avoid conception (generally referred to as “contraception”) might not administer hormones after conception (generally referred to as “emergency contraception”).

### 1.1 History

Over eighty years have passed since the first attempts to develop a method that interferes with the events subsequent to sexual intercourse.

Since 1920 it’s known that estrogen -when administered early- could interfere with pregnancy; the first case reported in the 1960s literature is about a raped girl in a presumably ovulatory period, who was submitted to estrogen administration [7]. Since then, an increasing number of women was treated with high doses of conjugated estrogens, until -in the early 70s- the combined administration of estrogen and progestin was proposed.

In 1972, Yuzpe, a Canadian researcher published the first experimental data, using the self-called method [8]; meanwhile, trials with only made progestin drugs began [9], and in the late 70s the intrauterine device between the forms of “emergency contraception” [10] was introduced; recently also the mifepristone or RU486 [11] have been used for this purpose.

### 1.2 Definitions

Even the definition itself has aroused a lot of controversies about the use of emergency contraception. There are at least two main schools of thought: those who believe abortion only occurs after embryo have implanted into the uterus (they state that the hormones administered before implantation can be considered “contraception”) and those who believe that also the interruption of the embryonic development, before implantation, represents an abortion (they claim that the hormones administered after the conception are “abortion”).

It’s very important to define the beginning of pregnancy; on one side, the principal scientific associations believe that it begins with the egg’s implantation [12–16]; in the early 70s the ACOG (American College of Obstetricians and Gynecologists) has redefined the concept of pregnancy; the term “pregnancy” refers to the period between the implantation of the embryo in the uterus and childbirth [17]. On the other side, other authors believe that pregnancy starts when oocity is fertilized, assuming that the embryo is “born” in that stage [18,19].

To better understand the difficulties rising from the definition of “contraception” we must remember that in humans (assuming an average duration of the menstrual cycle of 28 days) the normal length of pregnancy is 40 weeks, totaling 280 days, calculated from the starting day of the last menstrual period. Instead, considering the ovulation day, the normal duration of the conception’s product development is 38 weeks, equal to 266 days. In this case, therefore, the term “pregnancy” includes not only the period from fertilization to birth, but also the 15 days preceding ovulation, since the reference empirically detectable is the last menstrual period [20].

Considering these dissimilar points of view, a differentiation have to be made between the concept of “emergency contraception”, “interception” and “abortion”.

Emergency Contraception -according to the WHO and the ACOG- is defined as all these methods that can give to the women a no risky way to prevent an unwanted pregnancy after unprotected intercourse, or in case of contraceptive failure, when it is used within 120 hours from unprotected intercourse [16,21].

Instead, if we consider begin of the pregnancy when the egg is fertilized, the use of post-coital methods may fall under the definition of abortion [18,19]

Even as regards the definitions of abortion, there are different points of view.

The definition suggested by WHO considers as abortion both the pregnancy terminated within 20 weeks of gestation and a born weighting less than 500g fetus; in other words, the process of gestation is included between the period when the zygote attaches itself to the uterine wall (about 14 days after conception), and the time when the fetus is capable of surviving on its own [21,22,23].

Others consider abortion as any method that acts by interrupting the pregnancy from the moment the egg is fertilized [24,25,26].

Interception is different from abortion because of the moment in which the contraceptive methods can interfere with the blastocyst implanting in the uterus [27,28,29].

Following some authors, the term “interception” corresponds to the term “abortion” [30].

### 1.3 Drugs

As a general rule “emergency contraception” can be achieved using the drugs reported in the following table [Tab 1]. This reviews aims at studying the most recent drugs around which the controversy about the actual mechanism of action has developed.

## 2 Levonorgestrel (LNG)

The trade name of levonorgestrel is norlevo, levonelle, lonel

### 2.1 Introduction

Levonorgestrel is a second generation synthetic progestogen used as an active ingredient in some hormonal contraceptives; chemically, it is a hormonally active levorotatory enantiomer of the racemic mixture norgestrel. It is a progestin derived from 19-nortestosterone [31].

LNG represents the active isomer of norgestrel and is administered orally or delivered either via an intrauterine device or from subdermal implants [32].

The levonorgestrel-only pill is commonly used for emergency contraception (EC), a back-up method for contraceptive failure which a woman can use within a few days of an unprotected intercourse to prevent an unwanted pregnancy.

The standard regimen is to be administered within 72 h (can be extended to 120 h) of an unprotected intercourse, 1.5 mg either as a single dose or in two doses 12 h apart [33]. This regimen is estimated to reduce a woman’s risk of becoming pregnant by 57–93% [34–38].

As EC the levonorgestrel intrauterine system and cupper intrauterine device [39] can be inserted up to five days after unprotected intercourse.

The levonorgestrel intrauterine system presents a lower incidence of pelvic inflammation and irregular bleeding.

A disadvantage of this type of contraception is that it can’t be used in women with vaginal infections, sexual infections and pelvic inflammatory disease.

### 2.2 Mechanism of action

There is wide controversy about the possible mechanism throughout the LNG, used for emergency oral contraception, prevents pregnancy; this is still an open question [40–44].

We considered both Levonorgestrel impact on ovulation and on endometrium.

#### 2.2.1 Impact on ovulation

Hapanagama et al. [45] studied twelve healthy women with regular cycles.

They administed LNG in the fertil period that was identifyed considering changes in the hormones levels of oestrone - 3 - glucuronide (E3G), a urinary metabolite of oestradiol and LH, evaluated with a monitoring system that includes disposable test sticks and a hand-held monitor.

Six women received LNG in the first study cycle and placebo in the third study cycle; the remaining six women received placebo in the first study cycle and LNG in the third study cycle.

The second study cycle was a washout phase for all women during which they also received placebo.

Seven women had apparently normal ovulatory cycles after taking LNG; five of them took LNG on the day before the LH surge and one on the day of the surge. In the four women in whom the LH peak and ovulation was delayed, the LNG was taken within three days of the predicted LH peak; one woman did not ovulate at all despite having an LH surge two days after taking LNG.

This seven women, who apparently ovulated normally, had a reduced total luteal LH and a shortened luteal phase; in fact, basal levels of LH are essential for the normal secretory function of the corpus luteum.

This study suggests that LNG -taken immediately before ovulation- acts as an emergency contraceptive by delaying or preventing ovulation.

Durand at al. [46] studied fourty-five healthy women with regular menstrual cycles.

All participants were admitted during the first ten days of their menstrual cycle and after a control cycle, were randomly allocated into four different groups: Group A: 15 women received two doses of 0,75 mg LNG taken 12 hours apart, with the first dose given on the morning of day ten of the menstrual cycle; Group B: 11 women received the same dose of LNG immediately after positive LH detection in urine; Group C: 11 women received the same dose of LNG 48 hours after positive detection of urinary LH and Group D: 8 women received the same dose of LNG in the late of follicular phase LH- (3 +/− 1).

Transvaginal ultrasound and serum LH were performed daily since the detection of urinary LH until follicular rupture. In addition, endometrial biopsies were taken – from all participants- at the day 9 after the LH peak.

Twelve participants of Group A did not ovulate and three did not ovulate but presented a significantly shorter luteal phase with lover progesterone levels.

In Groups B, C and D no modifications were noted in follicular phase length between treatment and control cycle.

In Groups B and C, no significant differences on either cycle length or luteal progesterone and estradiol concentrations were observed, while in Group D the cycle length was normal but progesterone luteal levels were significantly lower.

As to endometrial histology, the author concluded that in both control and treated cycles, weren’t observed inflammatory reactions or other abnormal features.

Only twenty-four of the thirty-three biopsies were studied, because of insufficient tissue sample.

Marions et al. [47] studied twelve administered LNG-ECPs (0,75 mg twice, 12 h apart) to six women twice: the first time two days before ovulation; the second time two days after ovulation.

They evaluated both the urinary hormonal levels and on endometrial biopsy obtained six-seven days after LH surge.

LNG pre-ovulatory treatment inhibited the LH peak; the post-ovulatory treatment did not affect the cycle pattern, but the endometrium was normal only in three patients, out of phase in two and insufficient in one.

Subsequently Marions [48] administered the same treatment to seven women two days prior to ovulation (day -2; assessed by ultrasound).

Both the urinary hormones and the destiny of the leading-follicle were evaluated. The treatment caused either a delay or an inhibition of the LH peak in all subjects.

A significant delay in P4 levels and an initial suppression of E1 levels were also noted. The development of the leading follicle was either arrested or continued without signs of rupture.

This study indicates that, when used for EC, LNG administered prior to ovulation acts through an impaired ovulatory process and luteal function.

Croxatto et al. [49] studied fifty-eight patients presumed healthy and normally cycling and valuated the effects of the standard LNG regime (0,75 mg X 2, with a 12 hours interval between doses) or a single dose (1,50 mg) or placebo.

Participants were randomly assigned to three groups: one group received the first pill when the leading follicle reached a mean diameter of 12–14 mm (n=18); in the second group, it was given when the diameter of the follicle was 15–17 mm (n=22), the third group received the first pill when the follicle reached 18 mm.

As result, the spontaneous occurrence of either lack of follicular rupture or ovulatory dysfunction was rather high: up to 62% when given LNG-ECPs at a follicle diameter of 12–14 mm, 45% at 15–17 mm and 13% when the follicle diameter already reached > 18 mm.

Subsequently, Croxatto with Noè et al. [50] conclude that LNG-EC prevents pregnancy only when taken before fertilization of the ovum has occurred; they studied a cohort of women attending a family planning clinic for EC.

On the day of intake of LNG-EC and during five days follow-up, blood samples were taken for examination of luteinizing hormone, estradiol and progesterone concentrations, and vaginal ultrasound examinations were done for size of the leading follicle and/or corpus luteum. Of 388 women attending for LNG-EC, 122 women had intercourse on fertile cycle days according to ultrasound and endocrine findings.

At the time of LNG-EC intake, 87 women were in Days −5 to −1 and 35 women were in Day 0 (day of ovulation) or beyond. With the use of the probability of clinical pregnancy reported by Wilcox et al. [N Engl J Med 333 (1995) 1517–1521], expected numbers of pregnancies among the 87 and 35 women were 13 and 7, respectively, while 0 and 6 pregnancies, respectively, occurred.

Recently the International Consortium for Emergency Contraception (ICEC) in collaboration with the International Federation of Gynecologists and Obstetrics (FIGO) has updated its joint Statement “*How do LNG-only emergency contraceptive pills (LNG ECPs) work to prevent pregnancy?*”.

The statement concludes that inhibition or delay of ovulation is LNG ECPs principal and possibly only mechanism of action; they still added that “Review of the evidence suggests that LNG ECPs cannot prevent implantation of fertilized egg [51,52].

The Statement conclusion isn’t shared by other authors [44].

Mozzanega take in consideration and analyze the works [45–49] on which is based the Statement and he observe that only Marion’s series [47,48] supports the Statement directly; in all the others the evidences are strikingly contrasting.

In the patients in which LNG really delayed or inhibited ovulation [45,46] were at the beginning of their fertile period when they received ECPs and so were the patients [49] treated when the leading-follicle had a mean diameter of 12–14 mm; unprotected sex in any previous day did likely occur when they were still infertile and the risk of pregnancy was likely null.

At last, he conclude that the number of the patients is really small and that the sample is not representative.

#### 2.2.2 Impact on endometrium

As regard the LNG impact on endometrium, the attenction of scientific literature is direct to investigate the LNG effects on the glycodelina endometrial expression and on glycodelina serum concentrations, both during peri-ovulatory midcycle [53,54] and at the time of LH surge [53,55].

Glycodelin is the mayor progesterone-regulated glycoprotein that is secreted into uterine luminal cavity by secretory/ decidualized endometrial glands [56].

Absence of glycodelin in the uterus during periovulatory midcycle is consistent with an open “fertile window”.

So, human endometrium contains no detectable glycodelin during the peri-ovulatory midcycle; the first appearence of glycodelin in endometrium is observed three days after the LH surge and its significant increase five-six days after LH surge, at the opening of the implantation window [57].

Glycodelin inibits NK cell activity [58], monocytic cell chemotaxis [59], T-cell proliferation [60], and it induces T cell apoptosis [61] at the concentrations present in endometrial tissue and uterine fluid.

Glycodelin induced by local or systemic administration of progestogens may potentially reduce the fertilizing capacity of sperm in any phase of the menstrual cycle: it is the first endogenous glycoprotein that was found to potentially and dose-dependently inhibit binding of human sperm to the sona pellucida [62].

Durand et al. [53] examined serum glycodelin concentrations and endometrial expression during the luteal phase following oral administration of levonorgestrel (LNG) at different stages of the ovarian cycle.

She studied thirty women that were allocated into three Groups; Group 1 received LNG on days 3–4 before the LH surge, Group 2 received LNG at the time of LH rise and Group 3 received LNG 48 h after the LH surge.

Serum progesterone and glycodelin were measured daily during the luteal phase.

The treatment with LNG before the LH surge shortened by four days the lag period before appearance of glycodelin in serum during the luteal phase. Serum glycodelin levels steadily rose from day LH+ 2 to days LH+7–8 after which they declined.

In this way the hormonal EC with LNG, taken before the LH surge, alters endometrial glycodelin secretion in two important phases of the cycle: the first is in the fertile window during which an early increase of glycodelin secretion is interest for its antifertility activity; the second is in the phase of uterine receptivity in which reduced glycodelin expression may reflect weakened immunosuppressive microenvironment within the uterus at the time of implantation.

Subsequently Durand [54] studied thirty healthy sterilized women with normal ovarian function; each woman received LNG during the pre-ovulatory phase approximately two days before the LH surge.

Both LNG did not modify follicle rupture in 20 of 30 women and serum glycodelin concentrations significantly increased during the early and mid-luteal phases.

These results may represent an additional action of LNG in situation where the intervention did not interfere with ovulation.

Durand [53] and Palomino [55] studied the effects of LNG when its was administered on the day of LH surge without changes of the secretory pattern of glycodelin in serum in the luteal phase.

Last, but not last, Meng et al. [63] studied a considerable number of markers of endometrial receptivity in women after LNG oral or vaginal administration.

The two regimens of LNG caused either only minor or no alterations in markers of endometrial receptivity.

## 3 Mifepristone

The tradename of Mifepristone is Mifegyne.

### 3.1 Introduction

In 1980, Etienne-Emile Baulieu, working for the laboratories of Roussel Uclaf on progesterone derivatives discovered a potent anti-progestin, initially called RU-38486 (abbreviated in RU486) Mifepristone: is a steroid with affinity for the progesterone and glucocorticoid receptors, greater than that of natural compounds [64].

There are several uses of mifepristone. Administration of RU486 followed by the prostaglandin, misoprostol, has been used successfully in the medical termination of pregnancy.This is the best known application of this drug. However doses of Mifepristone are also effective as contraceptive [64]. Also single doses of the drug appear to be effective as emergency contraception [64–66], but it is only market in China [49,67, Tab 1].

### 3.2 Mechanism of action

The effect of Mifepristone is on progesteron receptor. These belongs to a family of nuclear receptors includes receptors for the steroid hormones (glucocorticoid, mineralocorticoid, androgen, estrogen, and vitamin D) as well as for thyroid hormones and retinoids. These receptors are ligand-activated transcription factors with domains for DNA binding, hormone binding, and transactivation [68].

Specifically PR type A and B even if have similar DNA binding activities, they have different functional activities which depend on the cell type [65,69]. Studies carried out in PR-A and PR-B knockout mice have shown that PR-A is important for fertility, ovulation and uterine receptivity and mediates the anti- proliferative effect of progesterone in the follicular endometrium phase; Instead PR-B mediates differentiation and development of the mammary gland [70].

In the presence of mifepristone, receptors adopt an inactive conformation recruits corepressors such as nuclear receptor corepressor and silencing mediator of retinoic acid and thyroid hormone receptor. The result is a loss of transcriptional activity [71].

The action of Mifepristone in terminating early pregnancy, combinating with misoprostol, is due to its effects on the glucocorticoid [72] and progesterone [64] receptors located in the uterus. Both misoprostol and mifepristone induce cervical dilatation. Glucocorticoid receptor blockade may result in an inappropriate cytokine response of the immune system [73].The dose to achieve abortion is 200mg [74].

The contraceptive use of Mifepristone is due to its uterine and ovarian effects [75–77]. Instead emergency post-coital contraception probably is primarily due to inhibition of ovulation rather than inhibition of implantation [78].

It’s important to remark that the effects of Mifepristone vary depending on when the drugs is administer and the dose given.

#### 3.2.1 Impact on ovulation

Administration of Mifepristone during the follicular phase of the menstrual cycle delays the estrogen rise and the luteinizing hormone (LH) surge. Specifically the treatment with 10mg prior to ovulation showed more a tendency to postpone ovulation [78].

The precise mechanism by which mifepristone affects the timing of ovulation is enigmatic. Infact Mifepristone has been shown not to alter the pattern of pulsatile secretion of LH [79,80] or pituitary responsiveness to gonadotrophin-releasing hormone (GnRH) [81].

The ability of mifepristone to perturb ovulation and FSH secretion is thought to be a result of the anti-progestogenic effect [78], even if seems that mifepristone significantly modified circulating levels of leptin. Evidences suggest that leptin is an important modulator of the timing of menstrual cycles. Infact in laboratory animals, leptin is known to regulate GnRH secretion via neuropeptide Y [82], and prevents suppression of gonadotrophin secretion [83]. In humans, leptin can regulate the nocturnal LH profile in the mid- to late follicular phase that precedes ovulation [84].

The adipocytes expression and release of leptin from adipose tissue is stimulated by glucocorticoids [85–87]. So Mifepristone, with is anti-glucocorticoid action, suppress leptin in fat. It was also demonstrated with cultured adipocytes [88]. There is the possibility that, in humans, suppression of circulating leptin concentrations following ingestion of mifepristone (10 mg) contributes to the subsequent and transient decrease in gonadotrophin secretion [76].

#### 3.2.2 Impact on Endometrium

When a sufficient dose of mifepristone (10,50,600 mg) is given immediately after ovulation will significantly inhibit endometrial development [89,90]. It will also inhibit the expression of endometrial markers of endometrial receptivity such as leukema inhibitory factor, integrins, and cyclooxygenase [91,92].

Particularly, a single dose administation of mifepristone produces several effects in the endometrium, myometrium and decidua. If implantation has occurred, the inhibition of transcription by the mifepristone-PR complex results in down-regulation of progesterone-dependent genes with decidual necrosis and detachment of products of conception [93]. It also directly promotes uterine contractions by increasing myometrial cell excitability, establishing gap junctions between cells and influx of calcium [94]. It also increases the release of prostaglandins by decidual cells [95].

Mifepristone may also affect tubal functions at high dose (200mg) after LH surge. The tubal microenvironment is of great importance; Infact the preimplanting embryo is exposed to a changing, hormonally regulated during its time in the tubal lumen. It is likely that in the Falloppian tube the secretion of local factors, such as cytokines, is regulated by steroidal hormones; The inhibition of progesteron receptor mediated by mifepristone may impair the normal secretion of these cytokines [96].

Furthermore progesterone regulates tubal transport in vitro [97]; so a too rapid or too slow tubal transport could also be expected to cause desynchronization between the embryo and the tube, or the blastocyst and the endometrium [98,99]. Infact animal studies have previously shown accelerated tubal egg transport after mifepristone treatment [99].

Even if it is sure that in human, progesterone receptor concentration increased in tubal cells after Mifepristone administration, nevertheless the mechanism of action of these regimens when used for emergency contraception is not fully explained [96,98].

So Mifepristone can be used as emergency contraceptive as abortive/interception drug depending on dose and time of administration.

## 3 Ulipristal acetate

The trade name of ulipristal acetate(UPA)/CDB-2914 is EllaOne.

### 3.1 Introduction

Some trials show an higher failure rates when levonorgestrel is taken 48 hours or more after unprotected intercourse [37,100] so in May 2009, ulipristal acetate(UPA) was approved by EMEA as a safe and effective method of emergency contraception for use up to 5 days after unprotected sexual intercourse, on the basis of different trials [101–104] that included more than 4000 women.

UPA is a selective progesterone receptor modulator that inhibits or delays ovulation in women presenting 48–120 hours after unprotected intercourse when levonorgestrel efficacy markedly wanes. UPA has been shown to prevent ovulation and then fertilization even after the luteinizing hormone surge has begun [105].

UPA is orally active and taken as a single dose. So UPA is well accepted by women despite the insertion of an intrauterine device that many women find it unacceptable [106]. The major of women experience normal menses within 7 days of the expected date. Glasier [101] studied 1696 women. They received emergency contraception within 72 hours of sexual intercourse (UPA, n=844; levonorgestrel, n=852).

There were 15 pregnancies in the UPA group and 22 in the levonorgestrel group. In 203 women who received emergency contraception between 72 hours and 120 hours after sexual intercourse, there were three pregnancies, all in the levonorgestrel group.

This trial shows that UPA is non inferior to levonorgestrel for emergency contraception. This finding accords with the results of an earlier trial in which ulipristal acetate was at least as affective as levonorgestrel when taken up to 72 hours after sexual intercourse [102].

For women who present on the fourth or fifth day after sexual intercourse, UPA provides significant prevention of pregnancy whereas levonorgestrel did not and so for use beyond 72 hours and up 120 hours [101].

### 3.2 Mechanism of action

UPA exerts its pharmacological activity by binding progesterone receptors to produce an anti-progesterone contraceptive effect on ovary and on endometrium (endometrial thickness). These effects vary according to the timing of drug administration during the menstrual cycle [107].

In addition to its effect, UPA binds to glucocorticoid and androgen-receptor. However, its capacity as an antagonist in binding to these receptors is markedly lower than its anti- progestational activity [108].

#### 3.2.1 Impact on ovulation

The first studies to understand how the progesterone plays a key role during ovulation were done on mice. Mice lacking progesterone receptor gene fail to ovulate due to a defect in follicular rupture [108,109].

The biological effects of UPA vary according to the time of menstrual cycle that the drug is given and the doses. Singles doses of UPA administered during the mid-follicular phase suppress leading follicle growth, causing a dose-dependent delay in folliculogenesis. At higher doses, a new leading follicle is often recruited [110].

The effect of UPA at different follicular diameters and in relation to the LH peak and ovulation was studied. When given prior to LH rise, UPA inhibited 100% of follicular ruptures. When the size of the leading follicle was at least 18mm, follicular rupture failed to occur within the 5 and 6 days following UPA treatment in 20 (59%) and 15 (44%) subjects respectively. Even on the day of the LH peak UPA could delay ovulation for 24 to 48 hours after administration. Taken together these studies demonstrate that UPA may have a direct inhibitory effect on follicular rupture. This allows UPA to be effective even when administered immediately before ovulation when LH has already started to rise, a time when use of LNG or Yuzpe is too late for ovulation inhibition [105].

Other authors studied pathways that modulate ovulation: gene expression profiling was performed using ovaries from mice subjected to gonadotropin induced superovulation in the presence and in the absence of UPA. Prominent among the genes that were down-regulated in response to UPA was endothelin-2, a potent vasoactive molecule. Endoteline-2 mRNA was transiently induced in mural granulosa cells of the preovulatory follicles immediately preceding ovulation. Furthermore, mice trated with an endothelin selective antagonists of endothelin receptor B exhibited a dramatic (> 85%) decline in the number of released oocytes [111].

Administering UPA before ovulation causes delayed follicle development and release, probably a result of suppression of estradiol levels. If the EC drug is taken during the LH peak, follicular rupture and ovum release may also be delayed [108].

#### 3.2.2 Impact on endometrium

During the latter part of the menstrual cycle, UPA’s affect may be attributed to its ability to decrease endometrial thickness [112].

UPA, given at high or repeated doses have an effect on endometrial histology that could theoretically impair implantation of a fertilized oocyte [113].

Glasier believes that the dose of 30 mg of ulipristal acetate [101] was specifically titrated for emergency contraception on the basis of inhibition of ovulation and might be too low to inhibit implantation.

Fifty-six women with regular cycles were studied by Stratton et al. [113], using a single dose of UPA (10,50 or 100 mg) or placebo after ovulation and within 2 days of the LH surge. Four to six days later, a transvaginal ultrasound scan measured endometrial thickness, and an endometrial biopsy specimen was obtained. The endometrium was evaluated by immunohistochemical analysis for progesterone (P) –dependent markers.

UPA caused a significant dose-dependent decrease in endometrial thickness, an increase in glandular P receptors, and a decrease in peripheral node addressins. Estradiol and P levels and menstrual cycle timing were not altered.

The endometrial development was considered abnormal if there was > 2 days delay of pathologic dating compared with the chronologic day of cycle. The chronologic day of cycle was determined as the number of days from ultrasound-documented ovulation to biopsy.

Similar endometrial effects have been observed among those receiving mifepristone [114,115].

UPA also was associated with decreased expression of peripheral node addressins, which are important L-selectin ligands found on the surface of endothelial cells. Recent studies have shown that L-selectin ligands are up- regulated during the implantation window, making the uterus more receptive to the trophoblast [116,117].

All these evidences would interfere with the implantation phase.

## 4 Discussions and conclusions

This review demonstrates that the hormonal products used in emergency contraception play different roles depending on the phase of the cycle during which they are administered. As things stand now, this conclusion allows to consider that in some cases the effect falls under hormonal contraception before the formation of the embryo and in some other cases the effect falls under the interruption of the development of an already formed embryo. Statistically, it is not possible to determine which is the dominant effect both because this evaluation depends on the exact knowledge of the ovulatory period of every woman when taking these hormones and because the studies carried on to date have not yet achieved a statistically significant number of cases.

It seems at any rate possible to consider that the discussion about these molecules may be a calm scientific debate on its biochemical and clinical aspects. A polemic about the possible harmful effects on the embryo does not seem helpful, since the international scientific community has already accepted the approach to the safe methods of the intentional abortion. This practice is indeed legal, safe and medically supervised in many countries. Finally, it is desirable that every woman is enabled to accurately know the effects of every drug at her disposal. In so doing she can knowingly choose whether using it or not only depending on her conscience and her needs.

## Figures and Tables

**Tabel 1:** Extract of “Dedicated Emergency Contraceptive Pills Worldwide”; avaible at www.not-2-late.com

**County**	**Levonorgestrel ECPs**	**Ulipristal Acetate ECPs**	**Mifepristone ECPs**
**Albania**	Norlevo 1,5mg Postinor2		
**Algeria**	Pregnon		
**Andorra**	Norlevo 1,5mg		
**Angola**	Optinor		
**Argentina**	Norgestrel-Max, Norgestrel Max unidose		
**Armenia**	Escapelle, Postinor		
**Australia**	Levonelle 1*, Norlevo1,5* Postinor*		
**Austria**	Postinor 1500*, Vikela*	ellaOne	
**Bahamas**	Optinor		
**Bangladesh**	Postinor-2		
**Belgium**	Norlevo1,5*, Postinor*, Postinor1500		
**Bolivia**	Gianique, ImediatN, Impreviat, Pilem, Postinor-2, Pregnon, Tace		
**Bosnia and Herzegovina**	Optinor		
**Botswana**	Pregnon		
**Brazil**	Diad, Minipil2, Nogravide, Pilem,Poslov Postino Uno, Postiner, Pozato, Pozato Uni, PPMS, Prevyol		
**Bulgaria**	Escapelle*, Postinor Duo*	ellaOne	
**Cambodia**	Pregnon		
**Cameroon**	Norlevo*, Optinor P2*		
**Canada**§	Norlevo 0,75**, PlanB**		
**Central Africa Republic**	Optinor		
**Chile**	Escapel-1, Escapel-2, Optinor, PostDay, Pregnon		
**China**	AiWuYu (0,75mg)* AnTing (0,75mg)* AnTing (1,5mg)* BaoShi Ting(Postinor-2,0,75mg)* DanMei (0,75mg)* DanMei(1,5mg)* HuiTing(0,75mg)* HuiTing(1,5mg)*, JinXiao(0,75mg)*, JinXiao(1,5mg)*, JinYuTing (15mg)*, KaRui Ting(1,5mg)*, Le Ting (0,75)*, NuoShuang (0,75mg)*, XianJu(1,5mg)*, Yi Ting(0,75mg)*, YuPing(0,75 mg), YuTing(0,75mg)*		Fu NaiEr(10mg)*, HouDingNuo(25mg), HuaDian (25mg),Si Mi An (25mg), BiYun (12,5mg)*, FuNaiEr(25mg), HouDing Nuo(10mg)*, HuaDian(10mg)*, Si MiAn(10mg)*
**Colombia**	Escinor 1,5mg, PostDay,Postinor-1, Postinor-2,Pregnon,Tace		
**Denmark**	Levonelle 1500, Norlevo 1,5 mg	ellaOne	
**Italy**	Levonelle, Norlevo 1,5mg, Unlevo 1500		
**Ireland**	Levonelle 1500, 1,5mg Norlevo		
**Lithuania**	Escapelle*, Postinor*	ellaOne	
**Luxemburg**	Norlevo 1,5*, Postinor*, Postinor 1,5*	ellaOne	
**Mexico**	Alterna, Glanique, Ladiades 0,75, Ladiades 1,5, Postday, Postinor-2, Postinor-2 Unidosis, Silogin 0,75mg, Silogin 1,5mg, Vika		
**Netherland**	Norlevo 1,5**, Postinor 1500**		
**Norway**	Norlevo 1,5** Postinor1,5 **	ellaOne	
**Portugal**	Norlevo 1,5*, Postinor 1500*	ellaOne	
**Russia**	Escapelle, Escinor 0,75, Escinor 1,5		
**Spain**	Norlevo 1,5*, Postinor*, Postinor 1500*		
**Sweden**	Norlevo 1,5*, Postinor 1,5*	ellaOne	
**Switzerland**	Norlevo 1,5 mg		
**United Kingdom**	Levonelle 1500, Levonelle OneStep*, Levonelle −1*, Levonelle −2	ellaOne	
**United States of America**§	NextChoice*, PlanB*, PlanB OneStep	ella	

* available directly from a pharmacist without a prescription

** available over the counter

§ Emergency contraception is available from certain pharmacists without a prescription in Alaska, California, Hawaii, M aine, Massachusetts, New Hampshire, New Mexico, Washington State, and Vermont. Currently, there are different age restrictions for the EC products available in the US. Next Choice and Plan B One-Step are available OTC to women and men aged 17 or older. ella is available only by prescription to women of any age.
